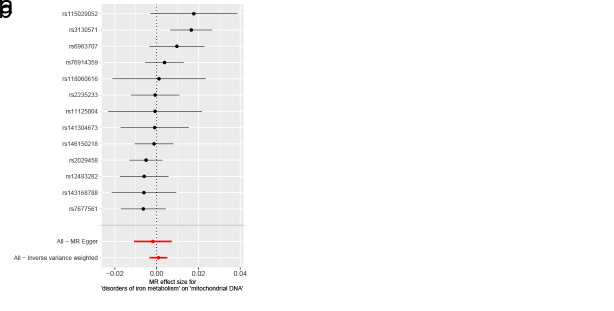# Mitochondrial related Mendelian randomization identifies causal associations between metabolic disorders and childhood neurodevelopmental disorders

**DOI:** 10.1097/MD.0000000000040481

**Published:** 2024-11-15

**Authors:** Chenyan Hu, Junjun Li, Pengfei Heng, Jianrong Luo

**Affiliations:** a Department of Laboratory Medicine, Medical Center Hospital of Qionglai City, Chengdu, Sichuan, China; b Department of Pharmacy, Sichuan Clinical Research Center for Cancer, Sichuan Cancer Hospital & Institute, Sichuan Cancer Center, Affiliated Cancer Hospital of University of Electronic Science and Technology of China, Chengdu, Sichuan, China; c Department of Laboratory Medicine, Institute of Traditional Chinese Medicine of Sichuan Academy of Chinese Medicine Sciences, Chengdu, Sichuan, China.

**Keywords:** autism spectrum disorder, childhood neurodevelopmental disorders, disorders of iron metabolism, disorders of lipoprotein metabolism, mitochondrial DNA

## Abstract

Childhood neurodevelopmental disorders (NDDs), including autism spectrum disorder (ASD), attention-deficit hyperactivity disorder, and Tourette syndrome, are a predominant cause of health-related disabilities in children and adolescents. Nevertheless, disease biomarkers are still limited. The aim of this study was to evaluate the potential, causal relationship between mitochondrial DNA copy number (mtDNA-CN), metabolic disorders, and childhood NDDs using the two-sample Mendelian randomization (MR) method. Genetic associations with mtDNA-CN, disorders of lipoprotein metabolism, and disorders of iron metabolism were selected as exposures, and genome-wide association data from ASD, attention-deficit hyperactivity disorder, and Tourette syndrome were utilized as outcomes. Results of the study suggested that a high degree of disordered lipoprotein metabolism related increases in ASD risk result from a decrease in mtDNA-CN (disordered lipoprotein metabolism–mtDNA: inverse variance weighting β: −0.03, 95% confidence interval: −0.05 to −0.02, *P* = 2.08 × 10^–5^; mtDNA-CN–ASD: inverse variance weighting odds ratio: 0.83, 95% confidence interval: 0.69–0.99, *P* = .034). The research findings implied that mtDNA-CN can mediate disorders of lipoprotein metabolism, potentially influencing the development of ASD. The potential impact of the results of this study for the prevention and treatment of childhood NDDs warrants validation in robust randomized clinical trials.

## 1. Introduction

Childhood is, in general, a period of fun, filled with sweets and games. For some families, however, it can be a time of severe health-related disruption. An estimated 53 million children and adolescents worldwide suffer from neurodevelopmental disorders (NDDs).^[1]^ Childhood NDDs are a group of chronic developmental disorders associated with brain dysfunction, including autism spectrum disorder (ASD), attention-deficit hyperactivity disorder (ADHD), and Tourette syndrome (TS). They have a variety of genetic and acquired etiologies.^[2]^ Even though children and adolescents with NDDs display a high level of resilience throughout the life course, they are more likely to experience academic underperformance, unemployment, and social isolation.^[3–5]^ This not only reduces their quality of life, but also leads to increased costs in health and welfare expenditures.^[6,7]^ Mounting evidence has indicated that genetic factors have a crucial role to play in the development of most childhood NDDs.^[8,9]^ Recently emerging technologies, especially genome-wide association studies (GWAS), have succeeded in identifying numerous novel biomarkers for childhood NDDs.^[10,11]^ Nevertheless, there is desperate need for a better understanding of the pathogenesis of childhood NDDs.

Mitochondria are complex multifunctional organelles that can participate in a variety of cellular functions, including calcium storage, heat regulation, reactive oxygen species production, and apoptotic signaling.^[12,13]^ On the basis of in vitro and in vivo mechanistic studies, mitochondrial dysfunction has been linked to an array of human diseases. Neuropsychiatric disorders are no exception with bipolar disorder, schizophrenia, and mood disorders all being linked to mitochondrial dysfunction.^[14]^ There are, however, a lack of precise biomarkers of mitochondrial dysfunction. Mitochondria possess a circular genome, composed of mitochondrial DNA (mtDNA), that is independent of the nuclear genome. The mtDNA copy number (mtDNA-CN) can indicate numerous facets of mitochondrial function.^[15]^ It has been observed that variations in mtDNA-CN may change the expression of mitochondrial genes and contribute to abnormalities in mitochondrial function.^[16]^ Particularly, mtDNA-CN has been found to be associated with mtDNA damage and oxidative stress, and thus may serve as a promising alternative biomarker for pathological conditions.^[17,18]^ However, the results of current observational studies on the relationship between mtDNA-CN and childhood NDDs are inconsistent. For example, several studies have reported that the presence of multiple mental disorders was associated with alterations in mtDNA-CN.^[19,20]^ Other studies, in contrast, concluded that there is no necessary association between mtDNA-CN and these mental disorders.^[21]^ In view of the typical limitations of small sample sizes and confounding factors in observational studies, none of these different findings reflects the relationship between mtDNA-CN and mental disorders with certainty. Therefore, more precise methods are needed to investigate the relationship between mtDNA-CN and childhood NDDs.

Metabolic disorders encompass a wide range of disorders in which toxic and/or complex compounds or energetic problems accumulate in cells due to enzyme or other protein dysfunctions. Recently, an increasing number of studies have demonstrated that childhood NDDs may be associated with inborn metabolic defects,^[22–24]^ such as disorders of lipoprotein metabolism and disorders of iron metabolism. As a critical component of the brain, lipids are involved in a multitude of physiological activities in the brain, including aiding structural development and neurogenesis and acting as signaling molecules.^[25,26]^ Disordered lipoprotein metabolism may provide a key mechanism in the etiology of different brain disorders, which include neuropsychiatric (including major depressive disorder and schizophrenia), neurodegenerative, and neurological disorders.^[27]^ Furthermore, iron (Fe), an imperative mineral nutrient for human health, plays an essential role in cellular energy metabolism and immunological function.^[28]^ Disorders of iron metabolism appear to be related to a variety of chronic diseases, including psychiatric disorders (Alzheimer disease and Parkinson disease).^[29]^ Several studies have demonstrated that ASD-associated inherited metabolic disorders can be mapped to metabolic disorders such as sterol biosynthesis, fatty acids, and metals through the involvement of differentially expressed microRNAs in metabolic pathways.^[30–32]^ However, current investigations have focused on a single disease, ASD, and there is a lack of systematic studies focusing on the relationship between childhood NDDs and metabolic disorders. Therefore, there is an urgent need for a comprehensive approach to validate the association of childhood NDDs with disorders of lipoprotein metabolism and disorders of iron metabolism.

Current mainstream experimental studies have mainly focused on directly exploring the effects of mtDNA-CN or metabolic disorders on childhood NDDs. Gu et al have found alterations in mtDNA-CN in the frontal cortex of patients with ASD.^[33]^ Rose et al have proposed that abnormal oxidative stress in children with ASD may damage mitochondrial DNA and induce disorders of lipid metabolism.^[34]^ These studies on the mechanisms of mtDNA-CN or metabolic disorders in the progression of ASD represent a promising area, but the results are still preliminary. In addition, the exploration of the pathogenesis of other childhood NDDs besides ASD is still in its infancy. Relevant epidemiological studies have yet to concurrently infer a causal relationship between mtDNA-CN, metabolic disorders, and childhood NDDs. A better understanding of the role between mtDNA-CN, disorders of lipoprotein metabolism, and disorders of iron metabolism may offer early opportunities for the prevention and treatment of childhood NDDs.

**Figure 1.**
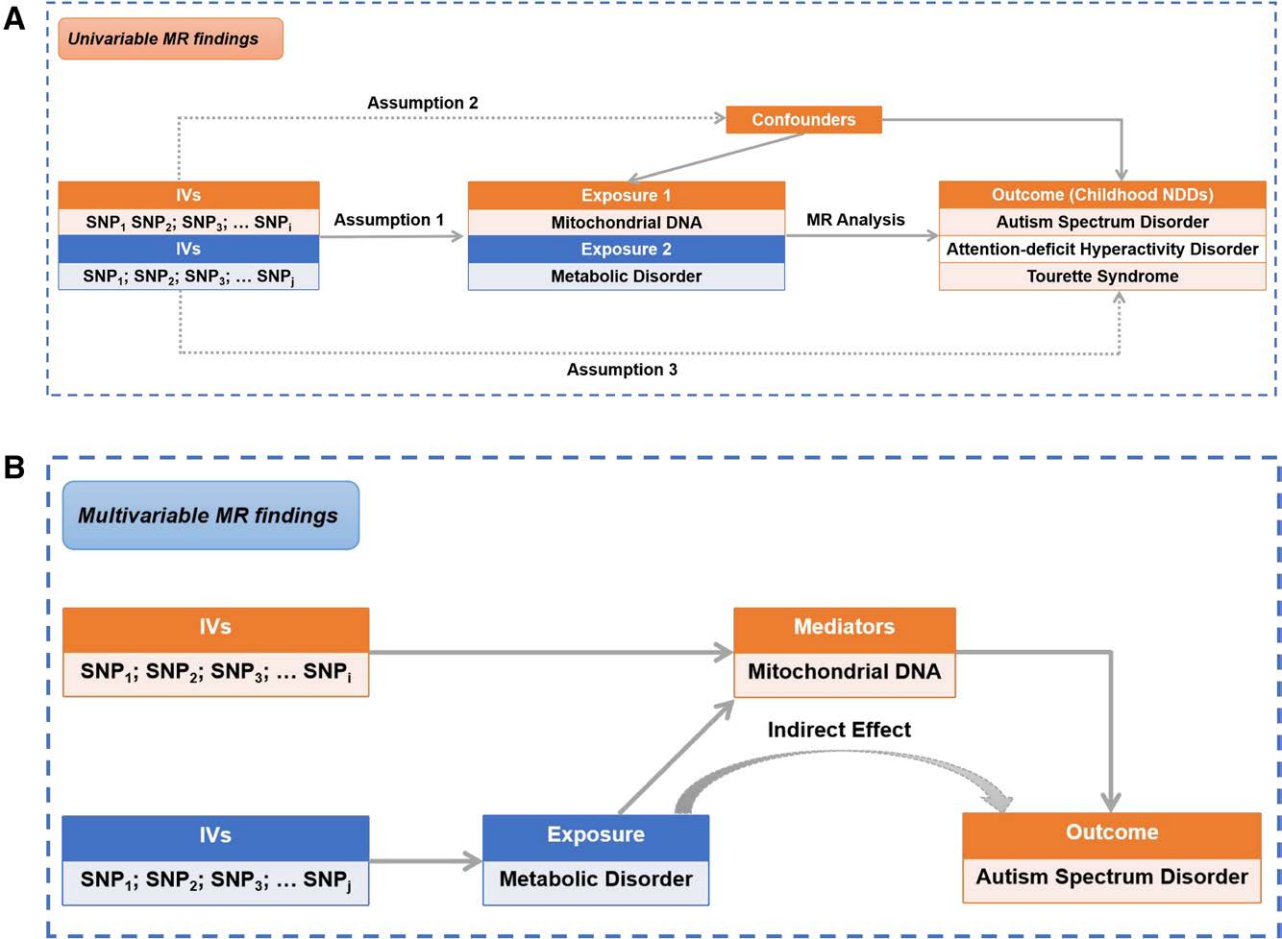
Graphical summaries of univariable (A) and multivariable (B) MR investigations. MR = Mendelian randomization; Childhood NDDs = childhood neurodevelopmental disorders.

Mendelian randomization (MR), an emerging approach to causal inference, infers whether exposures are potentially causal to outcomes by employing genetic variants as instrumental variables (IVs).^[35]^ MR analysis has the advantage of minimizing confounders and reverse causality bias due to the characteristic of inheritable and randomly allocated genetic variants.^[36]^ In this study, two-sample MR analysis was conducted to assess the potentially causal relationship of mtDNA-CN, disorders of lipoprotein metabolism and disorders of iron metabolism with childhood NDDs using summary statistics from large-scale GWASs (Fig. 1).

## 2. Methods

### 2.1. MR study design

In this work, univariable and multivariable MR were executed with summary statistics of GWASs from Chong et al,^[37]^ Psychiatric Genomics Consortium (PGC),^[38–40]^ and FinnGen consortium R9 release data.^[41,42]^ MR analysis, a genetically based analytical approach, utilizes the random allocation of genetic variation at meiosis and fertilization to infer the causal effect of exposure on outcomes. The single nucleotide polymorphisms (SNPs) employed as genetic instruments in the MR analysis must satisfy the following 3 basic assumptions^[43,44]^: (1) they should be robustly correlated with the exposure; (2) they should be independent of any potential confounds of exposure–outcome associations; and (3) they do not have a direct correlation with the outcome. More details of this method have been described in a previous study.^[45]^ This study adheres to the STROBE-MR guidelines.^[46]^ We first conducted two-sample univariable MR analyses to estimate the association of mtDNA-CN, disorders of lipoprotein metabolism, and disorders of iron metabolism with the risk of childhood NDDs (Fig. 1A). Multivariable MR was used to further assess the direct effect of disorders of lipoprotein metabolism and disorders of iron metabolism on childhood NDDs conditional on mtDNA-CN (Fig. 1B). Our analyses were not preregistered, so the results should be considered exploratory.

### 2.2. Data sources and instruments

Table S1, Supplemental Digital Content, http://links.lww.com/MD/N915 summarizes the original studies applied to this analysis and their sample sizes. The study depended on publicly available summary-level data and all original studies received ethical approval.

Data sources: in this study, summary-level statistics for mtDNA-CN were extracted from the recently published GWAS meta-analysis by Chong et al, consisting of 383,476 individuals of European ancestry.^[37]^ The summary results of the GWAS meta-analyses related to disorders of lipoprotein metabolism (N = 361,892, European ancestry) and disorders of iron metabolism (N = 324,474, European ancestry) were acquired from the FinnGen consortium R9 release data. GWAS summary-level data on childhood NDDs were all obtained from the PGC.^[38–40]^ GWAS summary-level data for both ASD (N = 46,351) and ADHD (N = 225,534) were acquired by combining samples from Integrative Psychiatric Research and PGC. The Integrative Psychiatric Research samples were gathered together from a population-based cohort of all children born in Denmark between 1981 and 2005. Meanwhile, the PGC samples were derived from several European cohorts. GWAS summary-level data for TS (N = 14,307) were acquired from PGC with samples from 4 European cohorts. All GWAS analyses on childhood NDDs, involving quality control, imputation, and primary association analyses were performed according to the PGC Ricopili GWAS pipeline.

Instrumental variables: to fulfill the first MR hypothesis, we selected exposure-related SNPs as IVs, with a significance threshold of *P* < 5 × 10^–8^ for mtDNA-CN and disorders of lipoprotein metabolism. For disorders of iron metabolism, the threshold was adjusted to *P* < 1 × 10^–5^ in order to capture more SNPs. Subsequently, linkage disequilibrium clumping was performed to identify independent SNPs (*r*^2^ < 0.001 within 10,000 kb) with the 1000 Genomes Project Phase 3 (EUR) as the reference panel. Assays were performed using the PhenoScanner GWAS database to determine whether the SNPs were potentially associated with outcome confounders (*P* < 1 × 10^–5^) (Table S2, Supplemental Digital Content, http://links.lww.com/MD/N916).^[47,48]^ The instrumental strength of exposure was assessed using 2 parameters, the proportion of variance (*R*^2^) explained by SNP and the F-statistic, with an F-statistic > 10 indicating that instrumental strength was adequate.^[49]^ The correlation was calculated as $R^{2}=\frac{Beta_{i}^{2}}{Beta_{i}^{2}+N\times {Se}_{i}^{2}}$ and $F=\frac{Beta_{i}^{2}}{{Se}_{i}^{2}}$,^[50]^ where Beta_i_ is the estimated genetic effect on exposure, Se_i_ is the standard error of Beta_i_, and N is the sample size. The effects of SNPs on exposure and outcome were harmonized, with genetic effects (Beta) and corresponding standard errors obtained. SNP palindromes with moderate allele frequencies were deleted and the residual SNPs were utilized for subsequent MR analyses. The genetic tools utilized in the MR analysis were listed in Tables S3–S5, Supplemental Digital Content, http://links.lww.com/MD/N917, http://links.lww.com/MD/N918, and http://links.lww.com/MD/N919.

### 2.3. Statistical analyses

Univariable MR: two-sample univariable MR was employed to evaluate the association of mtDNA-CN, disorders of lipoprotein metabolism, and disorders of iron metabolism with the risk of childhood NDDs. Two-sample MR analyses were conducted using the TwoSampleMR and MR-PRESSO packages in the R software (version 4.3.0; R Foundation for Statistical Computing, Vienna, Austria). The inverse variance weighted (IVW) method using a multiplicative random-effects model was applied to generate causal estimates as the primary outcome. IVW assumes that all IVs are valid; and yet, potential horizontal pleiotropy could introduce bias in the estimation.^[51]^ Therefore, a number of sensitivity analyses were carried out to resolve pleiotropy in causal estimation. The outliers in the selected IVs were first excluded with the MR pleiotropy residual sum and outlier (MR-PRESSO) method.^[52]^ The MR-Egger intercept test ascertained the presence of horizontal pleiotropy. MR-Egger results were prioritized if horizontal pleiotropy was affirmed (*P* < .05);^[53]^ otherwise, IVW results were deemed primary. Cochran *Q* test was adopted to detect heterogeneity, with a random-effects model of IVW for significant heterogeneity (*P* < .05), otherwise a fixed-effects model was used.^[54]^ Sensitivity analyses were conducted to address potential horizontal pleiotropy using MR-Egger, weighted median, simple mode, and weighted mode approaches. MR-Egger, which is sensitive to horizontal pleiotropy, reflects the average pleiotropic effect across all genetic variants through intercept estimates.^[55]^ The weighted median, on the other hand, allows for a certain percentage of invalid IVs, i.e., estimates are unbiased if at least 50% of the variables are valid instruments.^[56]^ Despite simple mode not being as robust as IVW, it is statistically capable of detecting pleiotropy.^[57]^ As for weighted mode, it yields a solid overall causal estimate when the majority of similar individual estimates are derived from valid IVs.^[58]^ Leave-one-SNP-out analysis was carried out to appraise the effect of association of individual variants on the estimate.^[59]^ Lastly, MR Steiger filtering tests were conducted to estimate the potential reverse causal effect of exposure on outcome.^[60]^ Results of MR analysis were expressed as odds ratio (OR) for binary outcomes or β for continuous outcomes with 95% confidence intervals (CIs).

Multivariable MR: to assess the direct effect of each exposure on the development of ASD, we carried out multivariable MR (MVMR) analysis, an extension of univariable MR permitting the joint detection of causal associations of multiple risk factors.^[61]^ Multivariable MR contemplates the relationship between metabolic disorders and mtDNA-CN, as well as the fact that SNPs utilized in MR analysis are generally correlated with metabolic disorders and mtDNA-CN (i.e., SNPs in our analysis were associated with metabolic disorders and mtDNA-CN with *P* < .05). Briefly, genetic instruments (metabolic disorders, mtDNA-CN) from relevant GWASs were combined and subsequently further clumped by a linkage disequilibrium analysis to ensure that the screened SNPs were independent (*r*^2^ < 0.001 within 10,000 kb). The IVW method was adopted to estimate the causal effect in the multivariable MR analysis. Multivariable MR analyses were conducted using the TwoSampleMR and MVMR packages in the R software (version 4.3.0; R Foundation for Statistical Computing, Vienna, Austria).

## 3. Results

### 3.1. Selection of tool variables

Univariable and multivariable MR analyses were performed to explore the causal effects of mtDNA-CN and metabolic disorders on childhood NDDs. Detailed information on all IVs associated with selected exposures after deletion of SNPs for incompatible alleles is shown in Tables S2–S5, Supplemental Digital Content, http://links.lww.com/MD/N916, http://links.lww.com/MD/N917, http://links.lww.com/MD/N918, and http://links.lww.com/MD/N919. The details of IVs for which F-statistics were calculated in all analyses are described in Tables S3–S5, Supplemental Digital Content, http://links.lww.com/MD/N917, http://links.lww.com/MD/N918, and http://links.lww.com/MD/N919. The F-statistics for all exposure-related IVs were >10. This demonstrated a low risk of weak instrumental bias in MR analyses.

### 3.2. Effects of mtDNA copy number on childhood NDDs

**Figure 2.**
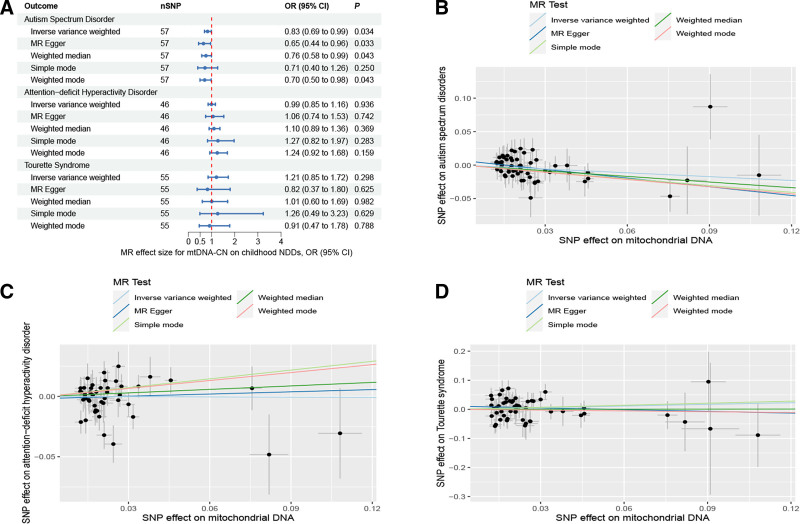
Mendelian randomization results of the association between mtDNA-CN and the risk of childhood NDDs. Sensitivity analysis results (A) of the causal relationship between mtDNA-CN and childhood NDDs. Scatter plots of the genetic risk of mtDNA-CN on ASD (B), ADHD (C) and TS (D) calculated with the inverse variance weighted (IVW), weighted median, MR-Egger, weighted mode, and simple mode methods. The slopes represent the causal relationship of each method. ADHD = attention-deficit hyperactivity disorder; ASD = autism spectrum disorder; Childhood NDDs = childhood neurodevelopmental disorders; CI = confidence interval; mtDNA-CN = mitochondrial DNA copy number; nSNP = number of single nucleotide polymorphisms; OR = odds ratio; TS = Tourette syndrome.

The results of univariable MR analysis robustly indicated a causally protective effect of mtDNA-CN on ASD development. A 1-standard deviation increase in mtDNA-CN was correlated with a 17% reduction in the risk of ASD (IVW OR: 0.83, 95% CI: 0.69–0.99, *P* = .034) (Fig. 2A and 2B). Similarly, the causal estimates were roughly aligned, when sensitivity analysis was conducted with other methods (MR-Egger OR: 0.65, 95% CI: 0.44–0.96, *P* = .033; weighted median OR: 0.76, 95% CI: 0.58–0.99, *P* = .043; simple mode OR: 0.71, 95% CI: 0.40–1.26, *P* = .250; weighted mode OR: 0.70, 95% CI: 0.50–0.98, *P* = .043). No potential outliers were detected by MR-PRESSO. Nor were horizontal pleiotropy or heterogeneity observed in the MR-Egger intercept test (*P* = .176), Cochran *Q* test (IVW-derived Q statistic = 42.2, *P* = .915) (Table S6, Supplemental Digital Content, http://links.lww.com/MD/N921), or the leave-one-out analysis (Figure S1, Supplemental Digital Content, http://links.lww.com/MD/N922). Furthermore, no evidence of reverse causality was found in the analysis of the MR Steiger test (Table S6, Supplemental Digital Content, http://links.lww.com/MD/N921).

Univariable MR estimates were not statistically significant for ADHD (IVW OR: 0.99, 95% CI: 0.85–1.16, *P* = .936) or TS (IVW OR: 1.21, 95% CI: 0.85–1.72, *P* = .298) (Fig. 2A, C, and D, Figures S2 and S3, Supplemental Digital Content, http://links.lww.com/MD/N924 and http://links.lww.com/MD/N925).

### 3.3. Effects of metabolic disorders on childhood NDDs

**Figure 3.**
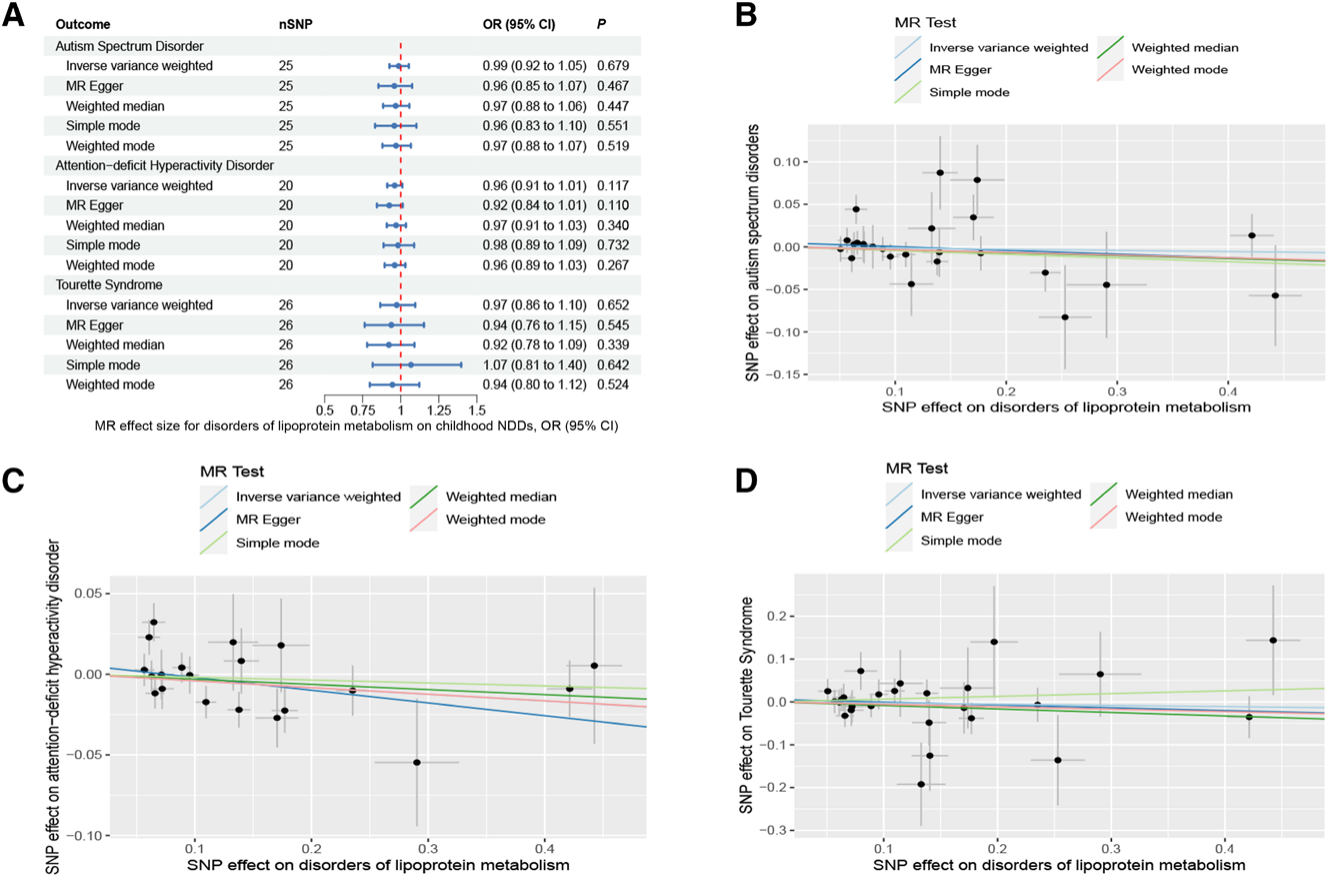
Mendelian randomization results of the association between disorders of lipoprotein metabolism and the risk of childhood NDDs. Sensitivity analysis results (A) of the causal relationship between disorders of lipoprotein metabolism and childhood NDDs. Scatter plots of the genetic risk of disorders of lipoprotein metabolism on ASD (B), ADHD (C), and TS (D) calculated with the inverse variance weighted (IVW), weighted median, MR-Egger, weighted mode, and simple mode methods. The slopes represent the causal relationship of each method. ADHD = attention-deficit hyperactivity disorder; ASD = autism spectrum disorder; Childhood NDDs = childhood neurodevelopmental disorders; CI = confidence interval; nSNP = number of single nucleotide polymorphisms; OR = odds ratio; TS = Tourette syndrome.

Lipoproteins and iron, participants in multiple physiological activities of the brain, whose metabolic disorders may result in irreversible damage to brain development in children and adolescents, could further contribute to the development of childhood NDDs.^[62]^ Accumulating evidence has now suggested, albeit inconsistently, an association between metabolic disorders and the development of childhood NDDs.^[63,64]^ Therefore, we conducted a two-sample MR study with metabolic disorders (disorders of lipoprotein metabolism and disorders of iron metabolism) as exposures and childhood NDDs as outcomes, in order to provide evidence of a causal relationship between metabolic disorders and childhood NDDs. When disorders of lipoprotein metabolism were employed as an exposure variable (Fig. 3), in the MR analysis of childhood NDDs, the univariable MR estimates were not statistically significant for ASD (IVW OR: 0.99, 95% CI: 0.92–1.05, *P* = .679), ADHD (IVW OR: 0.96, 95% CI: 0.91–1.01, *P* = .117) or TS (IVW OR: 0.97, 95% CI: 0.86–1.10, *P* = .652). Apart from ADHD, MR-PRESSO analysis for both ASD and TS did not detect any potential outliers. The MR-PRESSO result for ADHD exhibited the presence of a SNP (rs1169288) that interfered with the stability of the results. Following the removal of this SNP, it was found that disorders of lipoprotein metabolism had no effect on ADHD (Table S6, Supplemental Digital Content, http://links.lww.com/MD/N921). Additionally, the MR-Egger intercepts for all analyses were close to zero (*P* > .05), indicating that the MR results were not significantly affected by horizontal pleiotropy (Table S6, Supplemental Digital Content, http://links.lww.com/MD/N921). Cochran *Q* test revealed a lack of heterogeneity (*P* > .05). The leave-one-out analysis revealed that no outliers were found for the association between disorders of lipoprotein metabolism and childhood NDDs (Figures S4–S6, Supplemental Digital Content, http://links.lww.com/MD/N926, http://links.lww.com/MD/N928, and http://links.lww.com/MD/N929). The MR Steiger results supported that the included SNPs were more predictive of disorders of lipoprotein metabolism than childhood NDDs (*P* < .05) (Table S6, Supplemental Digital Content, http://links.lww.com/MD/N921).

**Figure 4.**
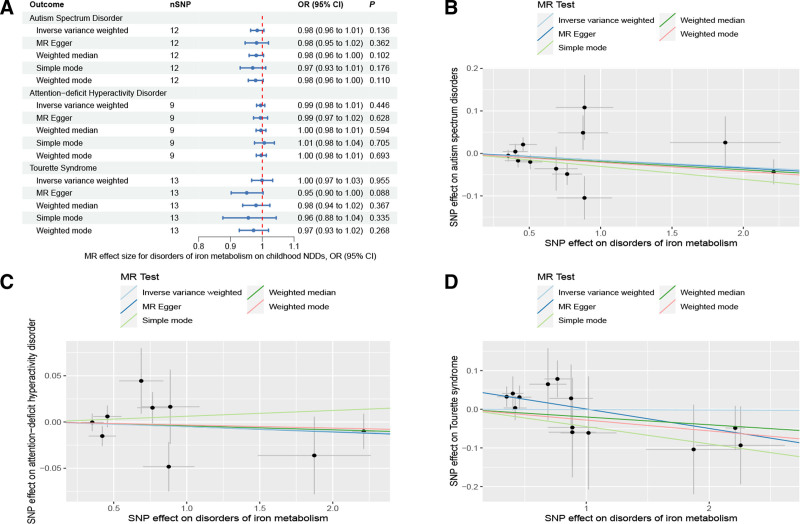
Mendelian randomization results of the association between disorders of iron metabolism and the risk of childhood NDDs. Sensitivity analysis results (A) of the causal relationship between disorders of iron metabolism and childhood NDDs. Scatter plots of the genetic risk of disorders of iron metabolism on ASD (B), ADHD (C), and TS (D) calculated with the inverse variance weighted (IVW), weighted median, MR-Egger, weighted mode, and simple mode methods. The slopes represent the causal relationship of each method. ADHD = attention-deficit hyperactivity disorder; ASD = autism spectrum disorder; Childhood NDDs = childhood neurodevelopmental disorders; CI = confidence interval; mtDNA-CN = mitochondrial DNA copy number; nSNP = number of single nucleotide polymorphisms; OR = odds ratio; TS = Tourette syndrome.

As for disorders of iron metabolism, effects on ASD (IVW OR: 0.98, 95% CI: 0.96–1.01, *P* = .136), ADHD (IVW OR: 0.99, 95% CI: 0.98–1.01, *P* = .446), and TS (IVW OR: 1.00, 95% CI: 0.97–1.03, *P* = .955) were not statistically significant (Fig. 4). Aside from TS, the MR-Egger intercept for both ASD and ADHD was close to zero (*P* > .05). The MR-Egger intercept test for TS provided evidence for the presence of horizontal pleiotropy (intercept: 0.051, *P* = .038) (Table S6, Supplemental Digital Content, http://links.lww.com/MD/N921). However, the MR-PRESSO of TS did not detect any potential outliers (*P* > .05) (Table S6, Supplemental Digital Content, http://links.lww.com/MD/N921). The Cochran *Q* test (*P* > .05) (Table S6, Supplemental Digital Content, http://links.lww.com/MD/N921) or leave-one-out analysis for all analyses (Figures S7–S9, Supplemental Digital Content, http://links.lww.com/MD/N930, http://links.lww.com/MD/N931, and http://links.lww.com/MD/N932) revealed no evidence of the presence of heterogeneity. The above results do not support a genetic relationship between metabolic disorders (disorders of lipoprotein metabolism and iron metabolism) and childhood NDDs.

### 3.4. Effects of metabolic disorders on mtDNA copy number

**Figure 5.**
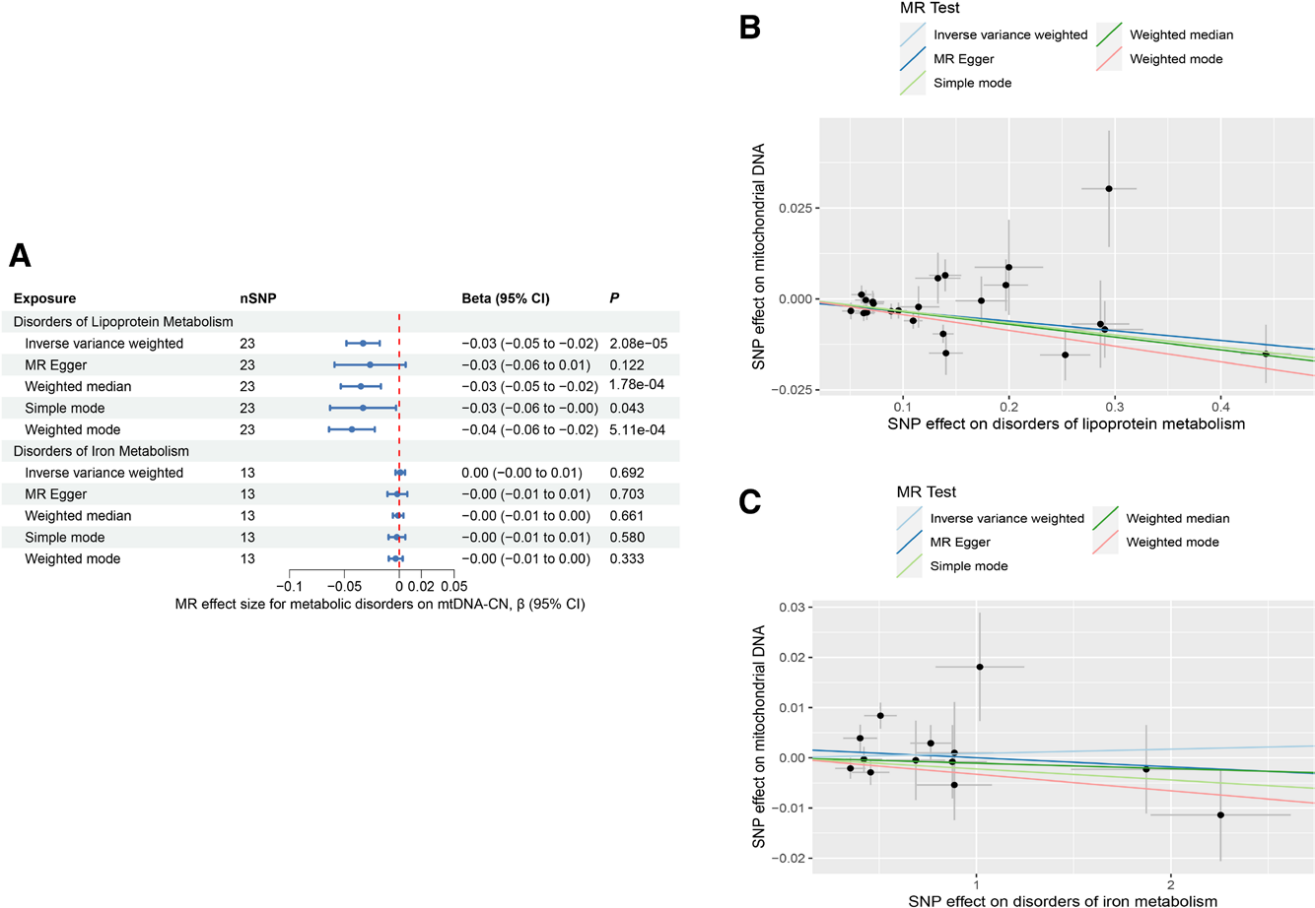
Mendelian randomization results of the association between metabolic disorders and mtDNA-CN. Sensitivity analysis results (A) of the causal relationship between metabolic disorders and mtDNA-CN. Scatter plots of the genetic risk of disorders of lipoprotein metabolism on mtDNA-CN (B) and disorders of iron metabolism on mtDNA-CN (C) calculated with the inverse variance weighted (IVW), weighted median, MR-Egger, weighted mode, and simple mode methods. The slopes represent the causal relationship of each method. CI = confidence interval; mtDNA-CN = mitochondrial DNA copy number; nSNP = number of single nucleotide polymorphisms; OR = odds ratio.

Given the crucial role of lipoproteins and iron in mitochondrial metabolism,^[21,65]^ we conducted univariable MR analyses in a two-sample framework to estimate the direct effects of disorders of lipoprotein metabolism and disorders of iron metabolism on mtDNA-CN. This revealed a causal relationship between disorders of lipoprotein metabolism and mtDNA-CN. An increase in disorders of lipoprotein metabolism was associated with a decrease in mtDNA-CN (IVW β: −0.03, 95% CI: −0.05 to −0.02, *P* = 2.08 × 10^−5^) (Fig. 5A and B). Disorders of iron metabolism, on the other hand, did not have a statistically significant effect on mtDNA-CN (β: 0.00, 95% CI: −0.00 to 0.01, *P* = .692) (Fig. 5A and C). The MR analysis of mtDNA-CN with disorders of lipoprotein metabolism as an exposure variable, the *P* values for the weighted median (β: −0.03, 95% CI: −0.05 to −0.02, *P* = 1.78 × 10^−4^), simple mode (β: −0.03, 95% CI: −0.06 to −0.00, *P* = .043) and weighted mode (β: −0.04, 95% CI: −0.06 to −0.02, *P* = 5.11 × 10^−4^) methods were significant. In addition, the causal estimates for MR-Egger (β: −0.03, 95% CI: −0.06 to 0.01, *P* = .122) were broadly consistent (Fig. 5A). The results of MR-PRESSO (Table S6, Supplemental Digital Content, http://links.lww.com/MD/N921) illustrated the presence of some SNP outliers (rs10455872, rs964184, rs13167071, and rs11536930) in this analysis. Nonetheless, a causal relationship was still found between disorders of lipoprotein metabolism and mtDNA-CN (Table S6, Supplemental Digital Content, http://links.lww.com/MD/N921) after removing these SNPs. For the horizontal pleiotropy analysis, the MR-Egger intercept *P*-value was >.05 (Table S6, Supplemental Digital Content, http://links.lww.com/MD/N921), suggesting that there was no horizontal pleiotropy present. The heterogeneity test identified an absence of heterogeneity (Cochran *Q*-*P*-value > .05) (Table S6, Supplemental Digital Content, http://links.lww.com/MD/N921). Leave-one-out analysis showed that MR analysis was reliable (Figures S10 and S11, Supplemental Digital Content, http://links.lww.com/MD/N933 and http://links.lww.com/MD/N934).

### 3.5. Multivariable MR findings

On the basis of all univariable MR results, we found evidence for a potential mechanism mediating how mtDNA-CN is involved in both metabolic disorders and ASD. Therefore, we subsequently investigated this mechanisms using two-sample multivariable MR analysis. In the MVMR analysis of disorders of lipoprotein metabolism–mtDNA-CN–ASD, the direct effect of disorders of lipoprotein metabolism on ASD had an OR of 0.97 (95% CI: 0.91–1.03, *P* = .275) after taking into account mtDNA-CN (Table 1). After taking into account disorders of lipoprotein metabolism, the direct effect of mtDNA-CN on ASD had an OR of 0.82 (95% CI: 0.69–0.98, *P* = .026). Similar to the univariable MR results, there was no direct causal effect of disorders of lipoprotein metabolism on ASD. However, the protective effect of mtDNA-CN against ASD endured. These results further support the hypothesis that disorders of lipoprotein metabolism can mediate mtDNA-CN to further influence the development of ASD. In the MVMR analysis of disorders of iron metabolism–mtDNA-CN–ASD, the effects of disorders of iron metabolism (OR: 1.01, 95% CI: 0.99–1.03, *P* = .322) and mtDNA-CN (OR: 1.17, 95% CI: 0.97–1.42, *P* = .094) on ASD were not statistically significant (Table 1). From the MVMR results, the pathway mediating the effect of mtDNA-CN from disorders of iron metabolism to ASD has yet to be identified.

**Table 1:** Multivariable MR analysis estimating the effects of mtDNA-CN and metabolic disorders on ASD.

Exposure	Autism spectrum disorder		
	nSNP	OR (95% CI)	*P*
Exposure 1			
mtDNA-CN	52	0.82 (0.69–0.98)	.026
Disorders of lipoprotein metabolism	22	0.97 (0.91–1.03)	.275
Exposure 2			
mtDNA-CN	51	1.17 (0.97–1.42)	.094
Disorders of iron metabolism	3	1.01 (0.99–1.03)	.323

ASD = autism spectrum disorder, CI = confidence interval, MR = Mendelian randomization, mtDNA-CN = mitochondrial DNA copy number, nSNP = number of single nucleotide polymorphism, OR = odds ratio.

## 4. Discussion

In this study, using genetic variants as unconfounded indicators of mtDNA-CN and metabolic disorders, we attempted to elucidate their association with the risk of childhood NDDs. To our knowledge, this is the first MR study to simultaneously assess the causal relationship between mitochondrial genomic features, metabolic disorders, and childhood NDDs. We found genetic evidence that mtDNA-CN play a mediating role in the effect of disorders of lipoprotein metabolism on ASD. Various MR methods demonstrated the findings to be largely robust. These methods impose different assumptions about horizontal pleiotropy, which suggests that horizontal pleiotropy is unlikely to adequately explain our results.

Since mtDNA-CN can indicate numerous aspects of mitochondrial function, an association of mtDNA-CN and childhood NDDs has been noted. This study showed that an increase in mtDNA-CN significantly reduced the risk of ASD, which is consistent with previous results.^[66,67]^ Singh et al observed the children with ASD have significantly lower copy numbers of key mtDNA genes that are involved in the functioning of the electron transport chain.^[66]^ Caporali et al, on the other hand, found that mtDNA-CN was significantly reduced in children with ASD compared to their normal age-matched siblings.^[67]^ Neurodevelopmental processes, including proliferation and differentiation of neural stem cells, migration and maturation of neurons, and formation and remodeling of neural synapses, rely on an adequate supply of energy.^[68]^ Mitochondria can produce ATP through oxidative phosphorylation, which provides energy for most cellular activities.^[69]^ Abnormal mitochondrial function may interfere with these processes, further affecting neuronal development and inducing dysfunction, increasing the risk of ASD.^[70]^ These findings emphasize the critical role of mtDNA-CN in ASD development. In addition, our study failed to find a significant association between mtDNA-CN and other childhood NDDs, including ADHD and TS. These MR results display inconsistent results with current observational studies.^[19]^ These discrepancies may stem from selection criteria, study design, sample size, mtDNA-CN measurement techniques, and potential confounders.^[71,72]^ Therefore, future studies should focus on thoroughly adjusting for confounders and selecting widespread prospective cohort studies to enhance study reliability.

However, univariable MR results do not support evidence that metabolic disorders have a direct effect on childhood NDDs. In MR analysis, etiological analysis usually involves statistical estimation of data with different MR analysis methods to detect and analyze small and medium effects.^[73]^ Our MR results on metabolic disorders contradict the results of current observational studies.^[63,64]^ However, there were also 2 studies that failed to observe any association between metabolic disorders and risk of childhood NDDs.^[74,75]^ Although there is a causal relationship between metabolic disorders and childhood NDDs as they are both associated with abnormal brain development,^[62]^ the results of MR analysis based on metabolic disorders do not support this relationship. To our hypothesis, this may be coincidental or confounded by some unidentified confounders. Furthermore, a causal relationship between metabolic disorders on childhood NDDs could not be ascertained in observational studies. A majority of patients with metabolic disorders tend to have a range of systemic diseases, and some individuals with childhood NDDs also suffer from several common diseases, such as obesity^[76,77]^ and diabetes.^[78,79]^ Thus, it is possible that some of the inflammatory pathways shared between these illnesses may result in a link between metabolic disorders on childhood NDDs.

Via two-step MR-mediated analysis, we demonstrated that lipoprotein metabolism disorders related increases in ASD risk result from a decrease in mtDNA-CN. In the first MR step, univariable MR ascertained a causal relationship between mtDNA-CN and ASD, with increased mtDNA-CN being associated with a reduced risk of ASD. Varga and colleagues reported that mitochondrial dysfunction caused by deletion of mtDNA is frequently associated with ASD.^[21]^ This is corroborated by our first MR estimate. The second MR step offered evidence that disorders of lipoprotein metabolism were correlated with reduction in mtDNA-CN. Consilient with this, it has previously been found that mitochondrial genetic variants are correlated with lipidomic analysis.^[65]^ However, there was a lack of correlative causality argumentation studies, and the two-steps of our mediation analysis precisely compensate for the deficiency. In addition, the results of multivariable MR further supported the existence of the disorders of lipoprotein metabolism–mtDNA-CN–ASD mediated pathway.

As 1 of the 125 scientific challenges plaguing basic scientific research, the etiology of ASD has become a major scientific problem requiring in-depth research both currently and in the future.^[80]^ According to relevant findings, we propose a hypothesis that mtDNA-CN mediates disorders the relationship between lipoprotein metabolism and the development of ASD. Abnormal reduction of high-density lipoprotein, an important component of blood lipids, is regarded as one of the most damaging disorders of lipoprotein metabolism.^[81]^ Previous studies have revealed that the decrease in high-density lipoprotein in patients with metabolic syndrome occurs concurrently with a decrease in mtDNA-CN.^[82]^ mtDNA is transiently attached to the inner mitochondrial membrane during mitochondrial oxidative phosphorylation. The massive amount of reactive oxygen species generated during this process damages the structure of mtDNA. Due to the lack of an adequate DNA repair mechanism in mitochondria, the reduction of mtDNA-CN and structural damage to mtDNA are anticipated to lead to severe defects in mitochondrial function.^[83]^ Normal functioning of the brain requires mitochondria to produce large quantities of ATP. Consequently, mitochondrial dysfunction (caused by mtDNA abnormalities) inevitably affects normal brain function, thereby affecting ASD progression.

The strength of this study is the comprehensive analysis of causal relationships between mtDNA-CN, metabolic disorders, and childhood NDDs in the two-step MR design based on IVs selected from large-scale exposure and outcome-related GWASs. Multiple sensitivity analyses were utilized to control for pleiotropy bias and to verify the robustness of the MR results. This study also has several limitations. First, while IVs associated with mtDNA-CN and disorders of lipoprotein metabolism were selected at a genome-wide significance threshold (*P* < 5 × 10^–8^), those associated with disorders of iron metabolism were selected at a relatively loose threshold (*P* < 1 × 10^–5^). This approach has been utilized in previous studies,^[84,85]^ but it comes with the limitation that it may lead to weak IV bias. However, the F-statistic was calculated to evaluate the risk of this bias and no strong evidence was found for its existence. We, nonetheless, still strongly recommend caution when interpreting our results. Second, the effect of disorders of iron metabolism on TS showed a significant MR-Egger intercept, suggesting a potential pleiotropy effect. To correct for potential pleiotropy effects, the MR-PRESSO analysis was performed and showed no potential outliers to be detected (*P* > .05). Third, the population genetic background analyzed by MR was limited to individuals of European ancestry, so further research is still needed in other ethnicities. Lastly, univariable MR results revealed that metabolic disorders did not directly affect childhood NDDs. However, the two-step MR-mediated analysis with mtDNA-CN as a mediator variable identified the presence of the disorders of lipoprotein metabolism–mtDNA-CN–ASD mediated pathway, which may be due to the fact that the mediator variable, mtDNA-CN, is a state rather than an trait variable. Therefore, our findings require further experimental validation and the mechanisms need further investigation.

Ultimately, the MR analysis conducted in this study, based on large-scale exposure and outcome-related GWASs, characterized the causal relationship between mtDNA-CN, metabolic disorders, and childhood NDDs. The research findings revealed that mtDNA-CN has a significant impact on the metabolic disorders–childhood NDDs pathway. Supportive evidence was provided that mtDNA-CN can mediate disorders of lipoprotein metabolism to further influence the development of ASD. The potential implications of the results of this study for prevention and treatment policies against childhood NDDs warrant validation in robust randomized clinical trials.

## Author contributions

**Conceptualization:** Chenyan Hu, Jianrong Luo.

**Data curation:** Chenyan Hu, Junjun Li.

**Formal analysis:** Chenyan Hu, Junjun Li.

**Investigation:** Chenyan Hu, Pengfei Heng.

**Software:** Chenyan Hu.

**Supervision:** Jianrong Luo.

**Visualization:** Chenyan Hu.

**Writing – original draft:** Chenyan Hu.

**Writing – review & editing:** Chenyan Hu, Jianrong Luo.

## Supplementary Material

**Figure:**
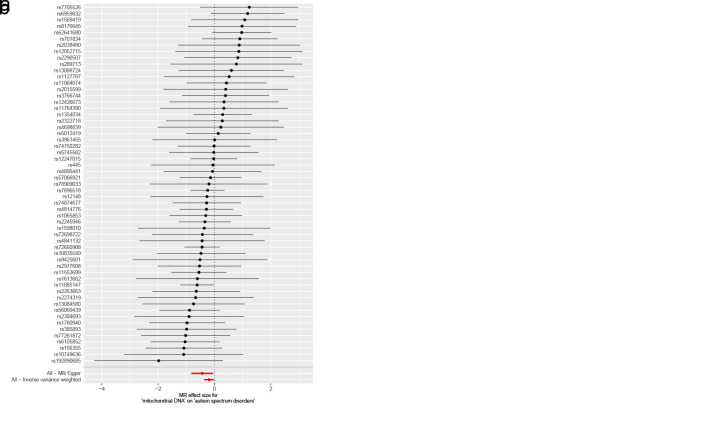


**Figure:**
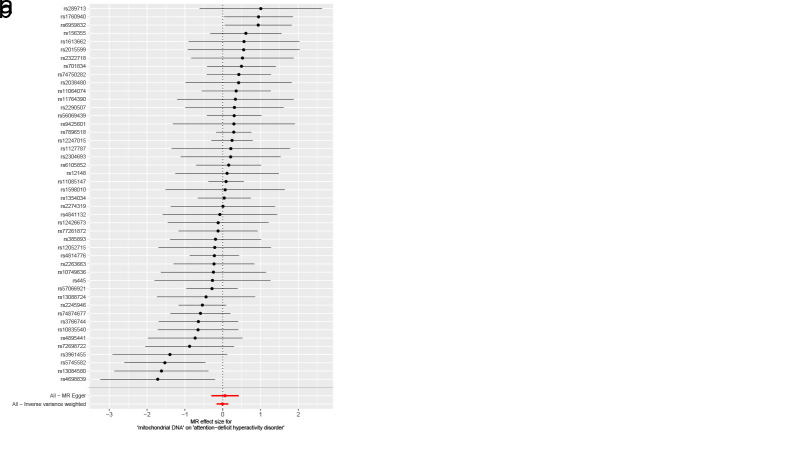


**Figure:**
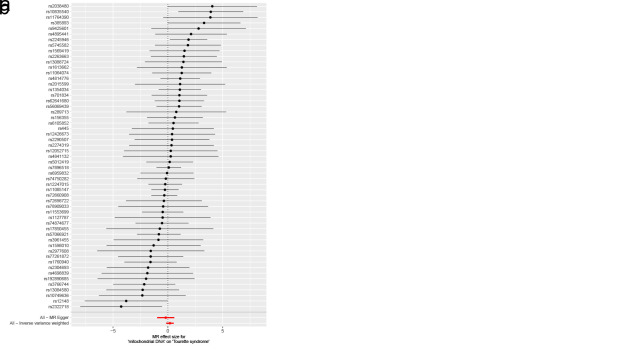


**Figure:**
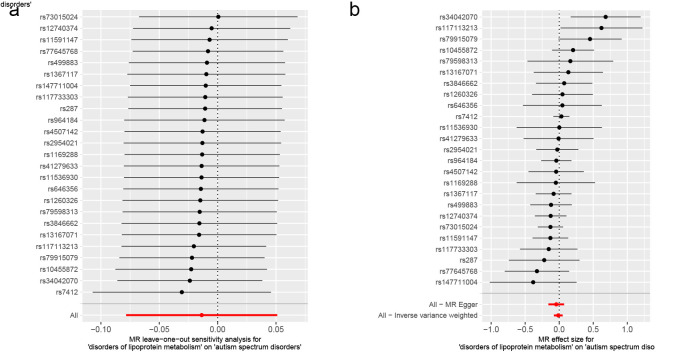


**Figure:**
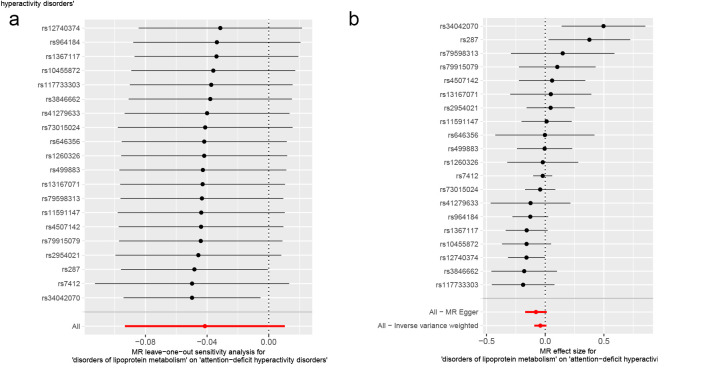


**Figure:**
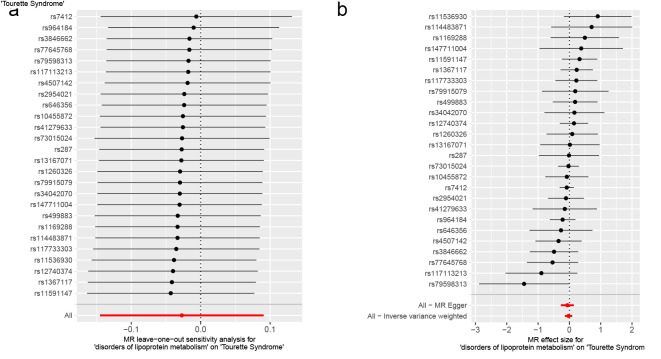


**Figure:**
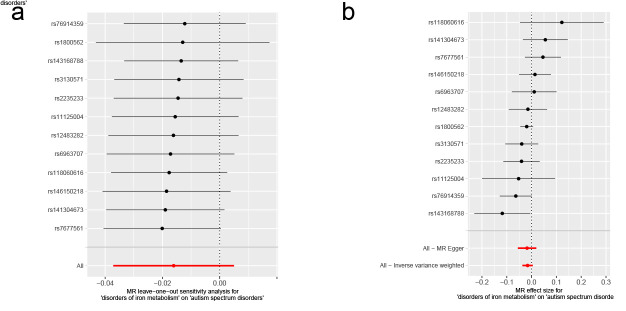


**Figure:**
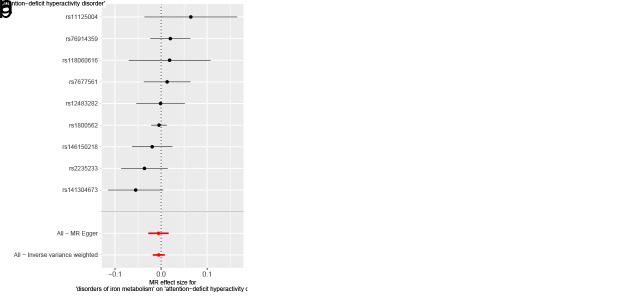


**Figure:**
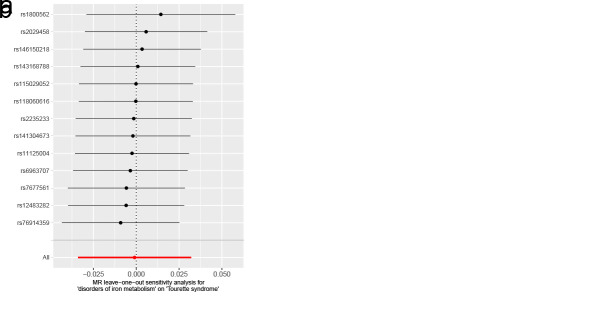


**Figure:**
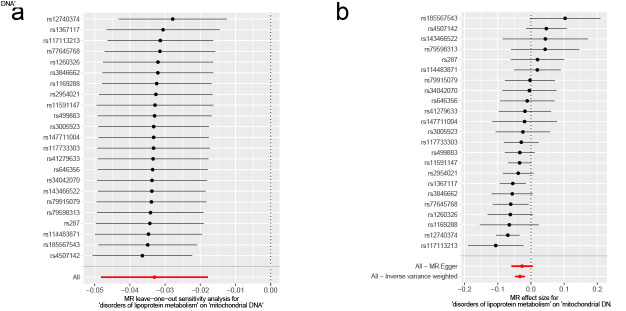


**Figure:**